# Immuno-based profiling of knee osteoarthritis patients identifies baseline characteristics associated with positive response to autologous platelet-rich plasma injection at one-year follow-up

**DOI:** 10.3389/fimmu.2026.1951258

**Published:** 2026-09-04

**Authors:** Valentina Granata, Antonio Tonutti, Veronica Marrella, Chiara Camisaschi, Simone Puccio, Cristiano Sconza, Marzia Monferini, Nicola Rani, Giuseppe Filardo, Berardo Di Matteo, Carlo Selmi, Emanuela Morenghi, Cristina Sobacchi, Angela Ceribelli

**Affiliations:** 1 Cellular and Humoral Innate Immunity Lab, IRCCS Humanitas Research Hospital, Milan, Italy; 2 Department of Biomedical Sciences, Humanitas University, Milan, Italy; 3 Rheumatology and Clinical Immunology, IRCCS Humanitas Research Hospital, Milan, Italy; 4 Milan Unit, National Research Council, Institute for Genetic and Biomedical Research (CNR-IRGB), Milan, Italy; 5 Immunity &Immunodeficiency Lab, IRCCS Humanitas Research Hospital, Milan, Italy; 6 Flow Cytometry Core, IRCCS Humanitas Research Hospital, Milan, Italy; 7 Lab of Translational Immunology, IRCCS Humanitas Research Hospital, Milan, Italy; 8 Department of Rehabilitation and Functional Recovery, IRCCS Humanitas Research Hospital, Milan, Italy; 9 Rizzoli-Argenta Orthopedics and Traumatology Unit, Rizzoli Orthopedic Institute, Bologna, Italy; 10 Faculty of Biomedical Sciences, Università della Svizzera Italiana, Lugano, Switzerland; 11 Division of Orthopedics, IRCCS Humanitas Research Hospital, Milan, Italy; 12 Biostatistics Unit, IRCCS Humanitas Research Hospital, Milan, Italy; 13 Bone Metabolism Unit, Laboratory of Translational Endocrinology & Metabolism, IRCCS Humanitas Research Hospital, Milan, Italy; 14 Rheumatology and Immunology Unit, ASST Spedali Civili, Brescia, Italy

**Keywords:** immuno-based profiling, inflammation, knee OA, oxidative stress, PRP injection

## Abstract

**Background:**

Osteoarthritis (OA) is a degenerative musculoskeletal disease of multifactorial origin causing chronic disability, despite numerous treatment options for which there are no predictive biomarkers on the different endotypes. We aimed at defining a peripheral blood (PB) profile potentially predictive of early and sustained response to intra-articular injection of autologous platelet-rich plasma (PRP).

**Methods:**

A prospective study was conducted on male and female patients aged 40–74 years, with Kellgren-Lawrence knee OA grade 2–3 candidates to PRP injection. PB was obtained from patients at enrollment prior to PRP injection to assess immune phenotype of PBMCs with multiparametric flow cytometry; serum antioxidant capacity; PBMC expression of molecules involved in inflammatory pathways; serum concentrations of related inflammatory cytokines and chemokines. After one year of follow-up, response was defined based on WOMAC questionnaires and clinical criteria and correlated with the immune profile.

**Results:**

Among 109 enrolled patients, 60 had early and sustained response to treatment and 49 were non-responders, irrespective of age, sex, and previous conservative therapy or surgery. Responders had lower oxidative stress and a peculiar profile of inflammatory markers (higher CD40L, Eotaxin 1; G-CSF, GROα, MIP3b, and PD-L1; lower TNFα, PTX3, and PDGF-AA) and immune cell composition (higher circulating CD14^+^; higher central memory CD8^+^, lower effector memory CD4^+^ and terminal effector CD4^+^ and CD8^+^ cells; lower Tregs) in the PB at baseline.

**Discussion:**

We describe an immunophenotype associated with early and sustained response to PRP treatment in knee OA and submit that this may be candidate to validation for predicting clinical benefit.

## Introduction

Osteoarthritis (OA) is the most common form of degenerative and chronic arthritis in adults, characterized by cartilage deterioration, impaired chondrocyte metabolism and differentiation, increased extracellular matrix degradation, and altered subchondral bone homeostasis. Its prevalence increases markedly with age, which explains the dramatically raised occurrence in the general (overall ageing) population in the last decades. Due to the increasing prevalence observed with ageing and the relevant symptomatic burden characterized by chronic pain, loss of mobility and disability, the prevention and early treatment of OA currently represent a clinical and sociodemographic priority, as recognized by the 2021–2030 agenda of the World Health Organization, in line with the general objective of promoting healthy ageing (1).

Among the different joints that can be affected by OA, the knee is the most frequent. The pathogenesis of knee OA is multifactorial and heterogeneous, including mechanical stress as in case of overweight or previous trauma, immune factors including both innate and adaptive mechanisms that mediate degradation and aberrant repair of the bone-cartilage complex, and ultimately genetic factors, with variants in cartilage structural genes, genes involved in the regulation of cartilage and bone development, DNA transcription, or in inflammatory pathways (2).

The treatment of knee OA is largely dictated by the clinical stage. Non-operative treatments are appropriate for early stages of disease and typically include weight reduction, physical activity, nonsteroidal anti-inflammatory drugs (NSAIDs), and repeated intra-articular injections of hyaluronic acid (HA) or corticosteroids (CS) (3–6). However, these therapies are limited in effectiveness, providing short-term symptom relief, and in the light of progressing disorder and disability, many patients finally require surgical interventions such as arthroplasty (7, 8). However, while prosthesis use in the middle-aged patient population is increasing over time, these patients present a double risk of failure with respect to older patients and, overall, results are often only partially satisfactory, which fueled research efforts towards new joint preserving strategies to postpone the need for arthroplasty to an older age (9). In this light, for patients who do not respond to standard treatments, are not candidates for surgery, or prefer to avoid surgery, thus orthobiologic therapies such as platelet-rich plasma (PRP) injection may constitute an option to address this treatment gap, as recently endorsed by scientific societies, expert consensus statements, and an increasing body of evidence (10–13).

PRP is an autologous blood-derived preparation containing high concentration of platelets (up to five-fold the concentration in the peripheral blood) and growth factors such as transforming growth factor-β (TGF-β), vascular endothelial growth factor (VEGF), hepatocyte growth factor (HGF), fibroblast growth factor (FGF), and insulin-like growth factor-1 (IGF-1) that may stimulate cartilage repair upon intra-articular injection. PRP injection is safe and overall effective for symptomatic relief of pain in mild/moderate OA, based on both preclinical evidence and clinical follow-up evaluations of highly variable duration, ranging from 3 to 24 months (14–16); however, differences in the protocol and tools for PRP preparation, in the treatment scheme (number of doses and frequency) and likely in patients’ endotypes, may affect the outcome (13). Indeed, the identification of patient-specific characteristics that predict response to PRP would allow best targeting this regenerative medicine approach (17).

We recently reported the baseline immunoprofiling of peripheral blood in a cohort of knee OA patients which were treated with PRP injection and divided in responders and non-responders at mid-term (6 months) follow-up (18). In detail, 55 patients had completed the 6-month follow-up and, among them, 45 were classified as responders, with higher serum levels of ascorbic acid and 4-Hydroxynonenal (4-HNE), higher inflammatory signature, including upregulation of interleukin (IL)-1, IL-6, and tumor necrosis factor (TNF) signaling pathways, increased Receptor Activator of Nuclear Factor Kappa-B Ligand (*RANKL*) and Secreted Frizzled-Related Protein (*SFRP3*) expression, higher frequency of CD4^+^ T cells expressing HLA-DR, CD95, and PD-1, and CD8^+^ T cells expressing CD25 and CCR7, compared to non-responders. Thus, this mid-term report suggested that a specific set up of innate and adaptive immunity, factors involved in bone and cartilage remodeling, and distinct features of oxidative stress characterize the peripheral blood of patients with knee OA with positive response to PRP treatment. On the other hand, all regenerative therapies work through a gradual healing cascade, whereby the substantial pain relief achieved in the first months may (or may not) translate into a more durable amelioration and delay disease progression.

Based on these observations, in the present work the full cohort was analyzed after 1-year follow-up. Specifically, the patients were reclassified according to the comparison between response at 6-months and at 1-year follow-up (as detailed in the Methods). The new subgroups of responders and non-responders were numerically comparable at this point and all data collected at baseline were compared among them. The new analysis delineated an immunoprofile associated with early and sustained outcome of PRP injection: response to treatment was confirmed to be associated with lower frequency of T effector cells and higher frequency of memory T cells in the peripheral blood, as observed at 6 months (18). On the contrary, this new report modified the conclusion related to the inflammatory status, with lower protein levels of several cytokines in responders than in non-responders. Based on this, a model for future validation as predictor of treatment response is proposed.

## Methods

The methods are described elsewhere (18); in detail:

### Patients

The enrolled patients displayed grade 2–3 knee OA according to Kellgren-Lawrence radiological classification, age between 40 and 74 years, body mass index (BMI) < 40 kg/m^2^; previous failure of at least one conservative therapy; Western Ontario and McMaster Universities Arthritis Index (WOMAC) pain score between 1.75 and 4. After enrollment, patients had 60 mL of whole peripheral blood drawn for the evaluations described below and for PRP preparation (using the nSTRIDE kit, Zimmer Biomet, USA, according to manufacturer’s instructions), and were subsequently treated with a single intra-articular injection of autologous PRP. A clinical follow-up was performed after 6 and 12 months: response to treatment was considered positive if the patients reported an improvement > 40% from baseline in WOMAC total score (19, 20), based on questionnaires (21) both at the 6- and the 12-month-follow-up. Conversely, patients were classified as non-responders if they never reported improvement or if they had benefit only at one time-point (not both).

The study was approved by the Ethical Committees of IRCCS Humanitas Research Hospital (Rozzano, Milan, Italy) and IRCCS Istituto Ortopedico Rizzoli (Bologna, Italy); both Centers conducted patients’ enrollment, while all the analyses described below were performed at IRCCS Humanitas Research Hospital.

### Sample preparation

Peripheral blood mononuclear cells (PBMCs) were isolated from fresh blood samples using the Lympholyte-H Cell Separation Medium (Euroclone) according to the manufacturer’s instructions. Then, 1 × 10^6^ PBMCs were suspended in 600 µL Trizol (Qiagen) for RNA extraction, while the remaining cells were frozen in fetal bovine serum (FBS, Euroclone) with 10% dimethyl sulfoxide (DMSO, Sigma) and stored at –80 °C until thawing and further processing for Fluorescence Activated Cell Sorting (FACS) analysis.

Blood samples for serum isolation were collected in serum separation tubes (BD) and centrifuged at 1600 rpm for 10 min at room temperature (RT). After separation, serum was stored in aliquots at – 80 °C until use.

### Gene expression analysis

RNA isolation was performed according to standard procedures; then, 500 ng of total RNA were retrotranscribed using the High-Capacity cDNA Reverse Transcription Kit (Applied Biosystems) according to the manufacturer’s instructions. Quantitative PCR was performed on a ViiA7 Real-Time PCR Detection System (Applied Biosystems) using the SsoAdvanced™ SYBR^®^ Green Supermix (Bio-Rad) following the manufacturer’s protocol. The *18S* gene was used as housekeeping; the sequence of all the primers used are reported in our previous work (13). The relative gene expression analysis of target genes was conducted following the comparative 2^ΔCt method, and the normalized gene expression was represented as arbitrary units (AU).

### Serological evaluations

Total antioxidant capacity, 4-Hydroxynonenal–Protein (4-HNE) adducts, and vitamin E were measured in the serum using dedicated ELISA kits (Merck, Clinisciences, Abbexa, Vinci-biochem) following the manufacturer’s protocol of each kit; absorbance was read at the wavelength indicated by each protocol on a Promega™ GloMax^®^ Plate Reader. TNFα, IL-6 and IL-10 were quantified using the Ella™ Automated Immunoassay System (Bio-Techne, Minneapolis, MN, USA), following the manufacturer’s instructions. A set of additional inflammatory markers was measured using the Multiplex Human XL Cytokine Luminex Performance Assay 46-plex and PTX3 was quantified using a dedicated Luminex Assay (all from Bio-Techne) following the manufacturer’s instructions; then plates were read on a Bio-Plex reader (Bio-Rad).

### FACS analysis and data processing with CRUSTY

After thawing, PBMCs were stained with the fluorochrome-conjugated antibodies listed in our previous work (18). Briefly, Live/Dead staining was performed with Zombie Aqua for 10 min at RT in the dark. After washing, cells were stained in FACS Buffer and Brilliant Violet buffer (1:1 ratio) for 15 min at RT in the dark, then fixed in 1% paraformaldehyde (PFA) in FACS buffer overnight. Samples were acquired on a FACS Symphony A5 Instrument; at least 100, 000 events were registered for the analysis. Flow Cytometry Standard (.fcs) 3.0 files were imported into FlowJo software v10 and analyzed by manual gating to exclude aggregates and dead cells, and to identify live CD45^+^ cells. The gating strategy is presented in **Supplementary Figure 1**. Data were then converted into comma-separated values (CSV) files and analyzed using web-based platform for high-dimensional cytometry analysis CRUSTY (22). Dimensionality reduction was performed using UMAP, while unsupervised clustering was conducted with Full-PhenoGraph (K = 150).

### Statistical analysis

Data are presented as number and percentage, if categorical variables, or median and range, if continuous variables. Differences between responders and non-responders’ group were evaluated with chi square test, with Fisher correction if necessary, for categorical variables, or through Mann-Whitney U test for continuous variables. No correction for multiple test was performed at this stage. Boxes and whiskers plots were adopted to represent measured analytes in the responders and non-responders’ groups. Association with responder status with patients’ characteristics and measured analytes was also explored with a logistic regression. In order to create a model, all independent variables with a p value under 0.1 in both univariable analyses were then submitted to a multivariable logistic regression, and the results were presented as odds ratio (OR) with their 95% confidence intervals (95% CI). We excluded from this process the variables related to gene expression as this analysis cannot be easily integrated in the clinical practice, while we aimed to define a very usable model; the final model was also presented with its ROC curve. Statistical significance threshold was set at 0.05; unadjusted p values are reported. All analyses were performed using GraphPad Prism v.10 and Stata version 18.

## Results

In the cohort of patients with knee OA, 109 were eligible for this study and completed the 1-year follow-up; among them, 60 responded to intra-articular PRP injection, while 49 did not, according to the defined criteria. There was no significant difference between responders and non-responders as for age, sex, and previous knee surgery, while median symptom duration was significantly longer in non-responders to PRP injection (median duration: 24 vs 12 months in responders, p=0.031) (**Supplementary Table 1**).

**Figure 1:**
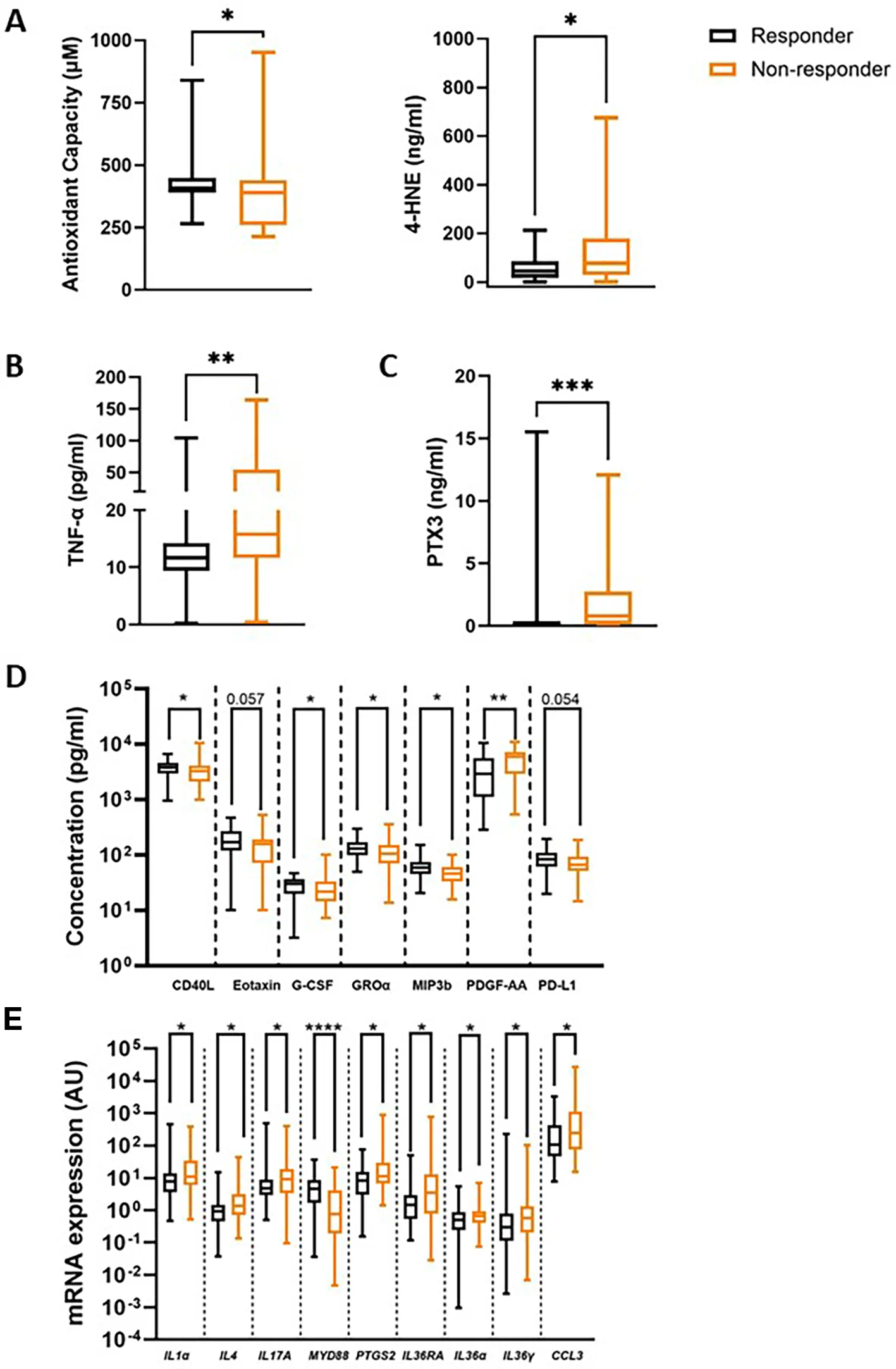
Evaluation of oxidative stress and inflammatory status in the serum collected at baseline from patients classified as Responders or Non-responders one-year after intra-articular PRP injection. **(A)** Serum oxidative stress status evaluated by ELISA assays. **(B)** Serum TNFα levels evaluated by ELLA system. **(C)** Concentration of PTX3 and **(D)** soluble markers including cytokines, chemokines, and growth factors measured by Bioplex System. **(E)** Gene expression analysis (qPCR) on the mRNA isolated from the patients’ PBMCs. Results are presented as mRNA levels, indicated as arbitrary units (AU), normalized on *18S* expression. In all graphs, data are shown as boxes and whiskers, representing medians and ranges; black boxes represent data from responders, while orange boxes those from non-responders. Significant differences from Mann-Whitney statistical test are indicated as asterisks. *p < 0.05, **p < 0.01, ***p < 0.001, ****p < 0.0001.

The serum level of total antioxidant capacity was significantly higher (number of subjects available for this readout: 60 responders, R, vs 49 non-responders, NR) and 4-HNE significantly lower in responders compared to non-responders (n=59 R vs 47 NR) (**Figure 1A**), confirming the observation (made at the 6-month follow-up) of higher antioxidant capacity and lower oxidative stress in the peripheral blood of responders to PRP injection.

The analysis of serum inflammatory markers showed that TNFα and PTX3 were significantly lower in responders compared to non-responders (n=52 R vs 23 NR for TNFα, and 58 R vs 47 NR for PTX3; **Figures 1B, C**). Moreover, CD40L, G-CSF, GROα, MIP3b, had significantly higher concentration in responders (n=60 subjects for all but GROα, where n=57) compared to non-responders (n=49 for G-CSF and MIP3b; 48 for CD40L; 42 for GROα). Eotaxin 1 (CCL11) and PD-L1 had a trend in this same direction (for both analytes, n=60 R and 49 NR), while PDGF-AA significantly lower concentration in responders (n=52 R vs 37 NR) (**Figure 1D**) and IL-6 did not differ in the two patient subgroups (n=52 R vs 23 NR; **Supplementary Table 2**). The exact median and range of all these immunological parameters, and p values for the comparison between responders and non-responders are shown in **Supplementary Table 2**.

Regarding gene expression of inflammatory pathways, we found significantly lower expression of *IL1α*(n=41 R vs 37 NR), *IL4* (n=57 R vs 48 NR), *IL17A* (n=56 R vs 47 NR), *PTGS2* (n=56 R vs 48 NR), *IL36RA* (n=56 R vs 49 NR), *IL36α* (n=57 R vs 48 NR), *IL36γ* (n=56 R vs 48 NR) and *CCL3* (n=58 R vs 49 NR), and higher expression of *MYD88* (n=58 R vs 49 NR), in the PBMCs of responders compared to non-responders (**Figure 1E**; **Supplementary Table 2**).

**Figure 2:**
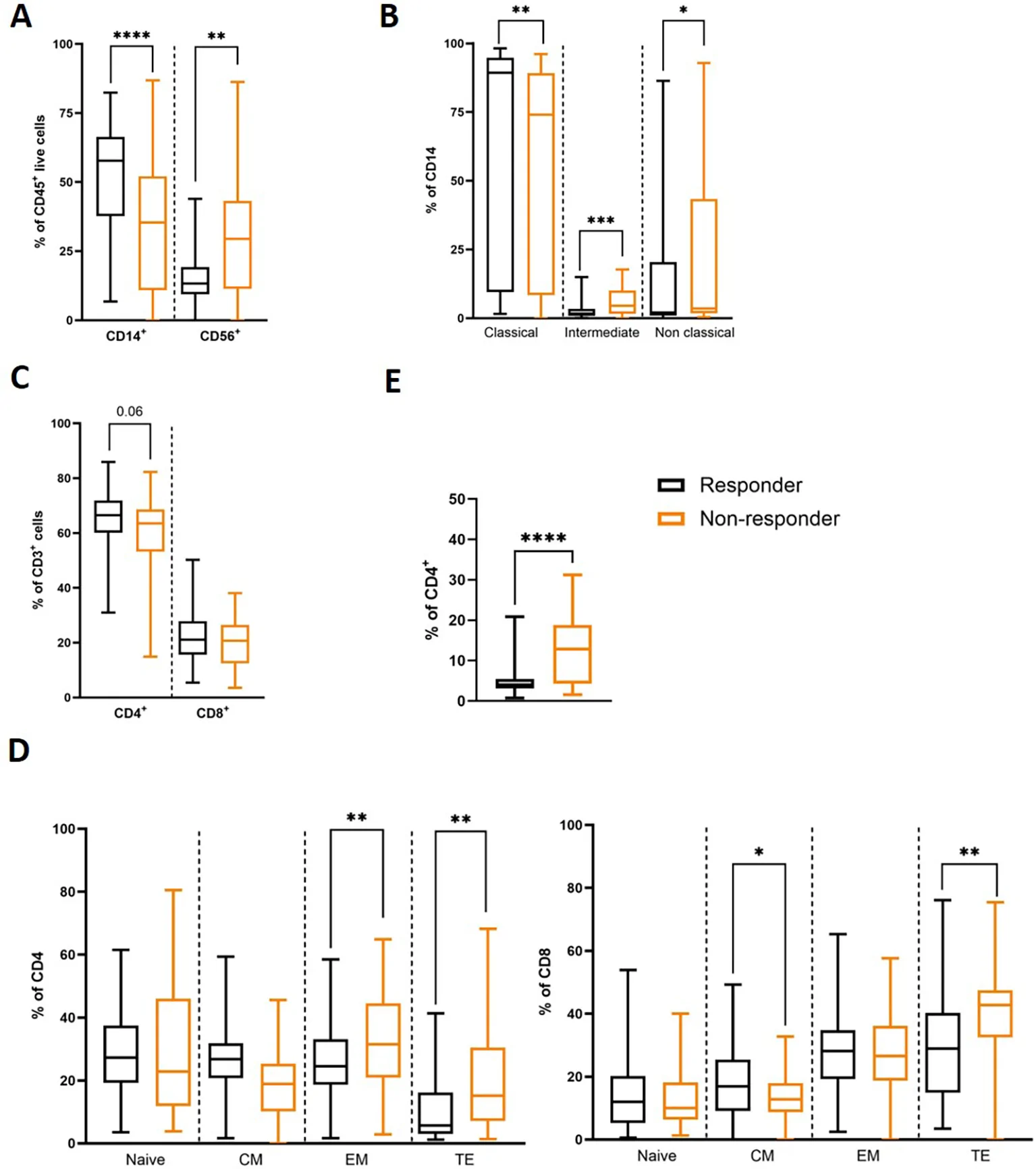
Multiparametric FACS analysis of PBMCs from responders and non-responders. **(A)** Frequency of CD14^+^ and CD56^+^ cell subsets, as percentage of CD45^+^ live cells. **(B)** Dissection of the CD14^+^ cell population in classical, intermediate and non-classical subsets. **(C)** Frequency of CD4^+^ and CD8^+^ cell percentage of CD3^+^ T lymphocytes and **(D)** distribution in naïve, central memory (CM), effector memory (EM) and terminal effector (TE) subsets. **(E)** Frequency of Treg cells. Data are shown as boxes and whiskers, representing medians and ranges; Mann-Whitney test. *p < 0.05, **p < 0.01, ***p < 0.001, ****p < 0.0001.

Multiparametric FACS analysis of the patients’ PBMCs revealed that responders had significantly lower frequency of NK (CD56^+^) cells and higher frequency of monocytes (CD14^+^ cells) (**Figure 2A**), particularly owing to higher classical monocytes (CD14^+^CD16^-^), while intermediate (CD14^++^CD16^+^) and non-classical (CD14^+^CD16^++^) monocytes were significantly lower compared to non-responders (**Figure 2B**). CD3^+^CD4^+^ and CD3^+^CD8^+^ cell populations did not differ clearly in the two subcohorts (**Figure 2C**), but notably in the CD4^+^ T cell subset effector memory (EM) and terminal effector (TE) cells were less abundant in responders, while in the CD8^+^ T cell subset central memory (CM) cells had higher frequency and TE cells lower frequency in responders compared to non-responders (**Figure 2D**). Finally, Treg cells (CD4^+^CD25^+^CD127^low/-^) were markedly less abundant at baseline in responders compared to non-responders (**Figure 2E**).

**Figure 3:**
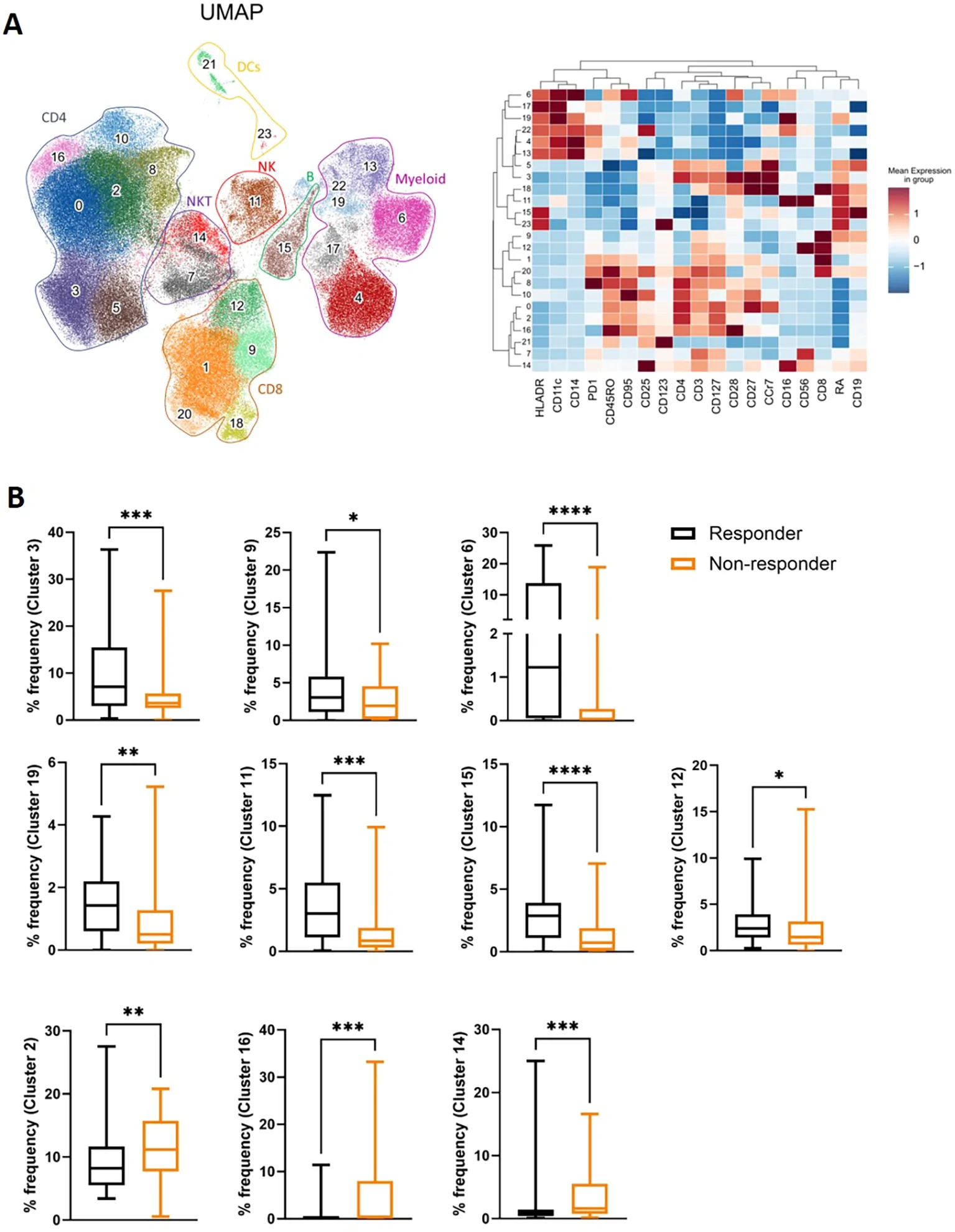
CRUSTY representation of FACS data. **(A)** On the left, UMAP dimensionality reduction. On the right, the immune phenotype of each cluster is summarized in the represented heatmap, with data z-scored column-wise for normalization. **(B)** Clusters more represented in responder and in non-responder patients. Data are shown as boxes and whiskers, representing medians and ranges; Mann-Whitney test. *p < 0.05, **p < 0.01, ***p < 0.001 ****p < 0.0001.

Unsupervised clustering analysis identified 23 different clusters of cells in responders and non-responders (**Figure 3A**). Seven clusters were more abundant in responders, namely clusters 3 and 9, corresponding to naïve CD4^+^ and CD8^+^ cells (both expressing CD45RA), respectively; clusters 6 and 19, corresponding to intermediate monocytes (both expressing CD11c, CD14, CD16, and HLA-DR); cluster 11, corresponding to NK cells (expressing CD16, CD56, CD45RA); cluster 15, corresponding to B cells (expressing CD19, CD45RA and HLA-DR); and cluster 12, corresponding to CD8^+^ cytotoxic cells (expressing CD56; **Figure 3B**). On the opposite, 3 clusters were more abundant in non-responders, namely clusters 2 and 16, corresponding to CD4^+^ cells with an effector or central memory phenotype (positive for CD45RO expression); and cluster 14, corresponding to NKT cells (**Figure 3C**).

Univariate and multivariate analysis were performed on serum parameters to identify baseline characteristics associated with response to PRP with easy clinical translation (**Supplementary Table 3**) and we found statistical significance for 4-HNE (odds ratio, OR 0.992; p=0.008), antioxidant activity (OR 1.004; p=0.029), Eotaxin (OR 1.003; p=0.089), GROα (OR 1.01; p=0.055; multivariate analysis p=0.004), PDGF-AA (OR 0.9998; p=0.008), TNFα (OR 0.97, p=0.013) and IL-6 (OR 0.88; p=0.040, multivariate analysis p=0.024).

**Figure 4:**
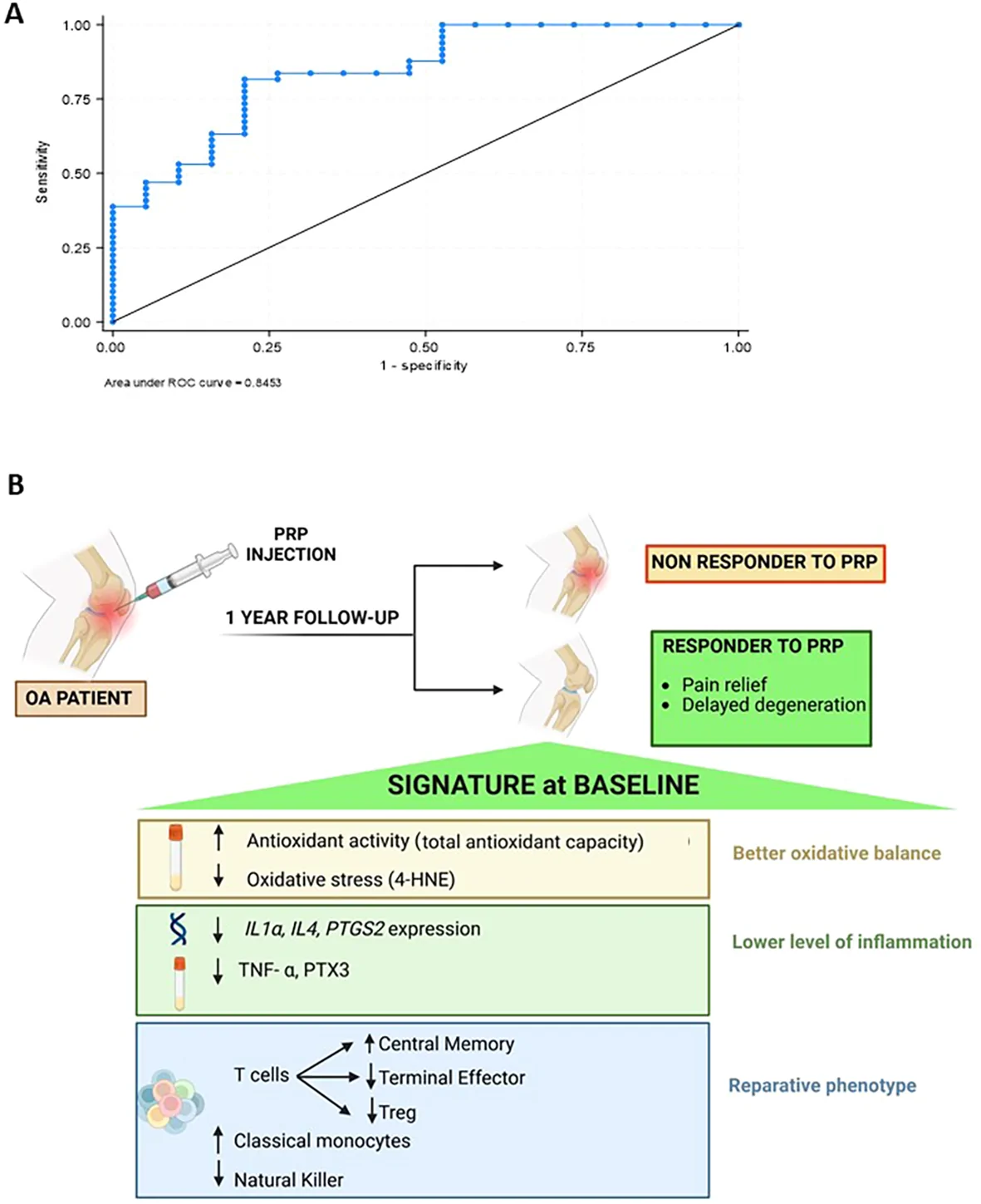
**(A)** ROC curve. **(B)** Summary of the study and immune-based profile suggested as predictive of positive response to intra-articular PRP injection in OA patients. Created with BioRender.com accessed on July 8th, 2026.

The parameters reaching statistical significance in the multivariate analysis were also used to define a ROC curve (**Figure 4A**) with an area under the curve (AUC) of 0.845 (95% CI: 0.742 to 0.948). In particular, for the final model the formula was: G2(x)=-1.15388 + 0.0278759* GROα -0.1987116* IL-6.

## Discussion

In the present work we describe the results of the evaluation of this cohort after a total of 109 individuals completed a 1-year follow-up. With this new analysis, we aimed at identifying an immunoprofile associated with (and thus possibly, predictive of) early and sustained response to PRP injection. In fact, there is growing evidence of a possible immunological and not only degenerative basis of OA, bringing into play pathways such as WNT and IL-1β-mediated signaling (23). In parallel, immunomodulatory capacity of the treatments available for knee OA must be considered, in particular with respect to orthobiologics, owing to their high content of cells and/or soluble factors able to enhance or dampen immune responses. In the case of PRP injection the exact mechanism of action is largely unknown, and it is not completely clear whether it has anti-inflammatory and immune-modulating functions (24) along with effects at both cartilage and synovial level (25). On the other hand, since PRP is an autologous blood-derived product, it sounds reasonable to speculate that the immuno-based profiling of the peripheral blood of patients (from which PRP is prepared) may allow identifying features that predict response to treatment.

We previously reported the multimodal profiling of peripheral blood from patients with early knee OA that received PRP intra-articular injection and were then evaluated after 6 months as for the amelioration of their condition (18). In that work, the analysis was conducted on 55 patients in total and revealed differences in terms of inflammatory and oxidative stress markers and lymphocyte populations between responders (45 individuals) and non-responders (10 individuals).

In the larger cohort described in the present work, 60 patients were classified as responders and 49 as non-responders; this latter subgroup comprised 4 subjects that had been classified as responders in the previous publication. Moreover, 4 patients (3 responders and 1 non-responder) included in our previous study were lost at the 1-year follow-up visit. Overall, the ratio between responder and non-responder after 1-year follow-up was lower than after 6-month (1.2/1 vs 4.5/1, respectively), likely owing to the stringent criteria applied for the classification as responder 1 year after PRP injection, including a single option, and the broader criteria for the classification as non-responder, including three different options (see Methods, Patients section). On the other hand, the high frequency of OA patients that do not experience any amelioration of their disease highlight the importance of a prediction tool with respect to use of time and resources.

As summarized in **Figure 4B**, in the 1-year follow-up cohort patients classified as responders had stronger antioxidant response capacity, as indicated by significantly higher levels of total antioxidant capacity and vitamin E, and lower oxidative stress, as indicated by 4-HNE, which is an index of lipid peroxidation. These findings agree with previous knowledge in the literature showing that oxidative balance is impaired in OA patients (26) and that OA severity has been directly correlated with the level of oxidative stress (27, 28). Furthermore, 4-HNE has been reported to be significantly increased both in damaged human cartilage and in a murine model of OA (29); and chondrocyte ferroptosis due to enhanced membrane lipid peroxidation has been proposed to contribute to OA progression (30). Our results suggest that responders to PRP injection have a better oxidative balance compared to non-responders. In fact, lower 4-HNE levels in responders point to lower ROS production; since oxidative stress is a highly modifiable factor, further investigations on the relation with OA and on its potential modulation aiming at the amelioration of response to orthobiologics could be considered.

We also analyzed several inflammatory markers in the serum, which had been previously quantified in diverse PRP preparations (31). We found that the levels of the long pentraxin PTX3, as well as those of TNFα, known to stimulate PTX3 release (32), were significantly lower in responders, suggesting a less inflamed milieu. Indeed, PTX3 is a member of the pentraxin family produced by immune and non-immune cells exerting functions in innate immunity, inflammation, tissue remodeling and bone metabolism, proposed as a predictor of osteoporosis and OA bone-related phenotypes (33). Enhanced expression of PTX3 was found in the synovium and articular cartilage of OA patients and OA-induced mice, and injection of exogenous PTX3 in the experimental model aggravated the phenotype by modulating macrophage reprogramming and exacerbating synovitis (34).

Moreover, higher levels of CD40L in responders are in line with higher frequency of CM CD4^+^ and CD8^+^ cells in this subgroup. Indeed, CD8^+^ T cells can express CD40L (35) and in turn CD40L stimulation enhances the number and memory phenotype of human peripheral CD8^+^ T cells (36). Higher levels of GROα (CXCL1) in responders were concordant with higher frequency of intermediate monocytes in this subgroup, which indeed can produce CXCL1 that stimulates migration of monocytes to injured or inflamed tissues. Also, Eotaxin 1 is strongly involved in the modulation of immune cell migration by binding to different CCRs (37). Findings in relation to OA are contradictory: Eotaxin-1 levels in the synovial fluid, but not in the plasma, have been positively related to the disease severity in knee OA patients of Han Chinese descent, and associated with the degree of tissue degeneration (38, 39); while in another paper Eotaxin 1 concentration was significantly lower in the synovial fluid of OA patients versus controls (40). Overall, this might point to stage- and site-specific functions of this chemokine, with a possible predominant role in the regulation of immune cell recruitment in our cohort of early OA patients.

G-CSF has immunomodulating, anti-apoptotic and anti-inflammatory properties, among others, and accordingly intra-articular injections have been tested in conjunction with activated autologous peripheral blood stem cells in early OA and gave promising results (41). Therefore, higher G-CSF plasma levels in responders compared to non-responders to PRP may be additional evidence of lower inflammation as a distinct characteristic of the formers.

Finally, we underline that besides the role in inflammatory processes, several molecules that we assayed in the sera of our patients also participate in immune cell recruitment, tissue repair, angiogenesis, and the resolution phase of inflammation (42, 43). Their increase in responders may therefore reflect a more responsive and regulated immune system capable of mounting a controlled reparative response following PRP administration rather than chronic inflammatory activation. On the same line, the lower levels of PDGF-AA in responders may indicate reduced baseline activation of profibrotic or maladaptive remodeling pathways, potentially allowing PRP-derived growth factors to exert greater biological effects. Interestingly, in the same family of proteins, the PDGF-BB isoform has been shown as a positive predictor for clinical response to autologous platelet concentrate therapy in grade I knee OA (44). This apparent discrepancy between the two protein isoforms could be easily explained by their functional differences (e.g. distinct biological compartments, receptor binding specificity and effects) and by differences in the patients’ cohorts investigated (e.g. disease stage).

In the gene expression analysis of PBMCs, overall, the signature of responders to PRP supported the interpretation that they had lower levels of inflammation. Interestingly, in a previous paper the rs20417G/C polymorphism in the promoter of the *COX-2* (alias *PTGS2*) gene was reported to contribute to the genetic risk for end-stage hip and knee OA; in particular, the C allele was associated with lower risk and the G allele with higher risk of end-stage OA (45). Moreover, the C allele was associated with lower promoter activity and lower levels of inflammatory plasma markers in patients undergoing coronary artery bypass graft surgery (46). Altogether, this evidence agrees with our interpretation of the findings in our study. We haven’t performed genetic investigations in our cohort of patients, so we don’t know which is the allelic frequency of the *COX-2* rs20417 variants in our study population, but we can foresee molecular analyses in future developments of this research.

As for IL36RA, it has been published that after DMM (destabilization of the medial meniscus) surgery (a preclinical model for OA), wild-type mice showed a decrease in IL-36Ra and an increase in IL-36α, IL-36β, IL-36γ, and IL-36R within the regions of increased mechanical loading (47), indicating that, in articular cartilage, the abnormal mechanical loading caused by destabilization of the medial meniscus may trigger downregulation of IL-36RA and upregulation of IL-36/IL-36R to induce OA progression. Accordingly, in human cartilage, OA severity was associated with decreased IL-36RA and increased IL-36α (47). In our dataset, the gene expression of *IL-36RA, IL-36α, IL-36γ* was lower in the PBMCs of responders than in non-responders, while we do not have information related to protein levels *in situ*; on the other hand, we might speculate that the condition where both IL36RA and IL36 are reduced corresponds to an earlier stage of disease.

*CCL3* and *IL17A* genes were also less expressed in the responders’ vs non-responders’ PBMCs. These findings fit with lower inflammation and lower T cell activation in responders than non-responders and is in line with the literature, where plasma CCL3 levels were significantly related to disease severity and discriminated between pre-X ray-defined knee degeneration (preX KD) and controls, on one hand, and between preX KD and severe OA, on the other (48); and IL17 levels in the serum (and synovial fluid, as well) were positively correlated with OA radiographic features (49).

Finally, in our gene expression analysis we found only one upregulated gene in responders vs non-responders, i.e., *MYD88* (as observed in the 6-month follow-up), which is in line with higher frequency of monocytes (CD14^+^ cells) in this subgroup, revealed by FACS analysis. In fact, at cytofluorimetric analysis several other immune cell populations were differently represented at baseline in responders vs non-responders, namely responders had also lower NK (CD56^+^ cells); markedly lower Treg cells; significantly higher CM CD8^+^ cells and lower EM and TE CD4^+^ and TE CD8^+^ cells. These observations suggested that OA patients who responded to PRP injection exhibit a “reparative” endotype at baseline. -Indeed, the increased frequency of classical monocytes in responders might reflect differences in the circulating monocyte pool available for recruitment and subsequent differentiation within inflamed tissues into macrophages or also dendritic cells (DCs) (50). In fact, monocyte differentiation and functional phenotype of monocyte-derived cells are strongly influenced by the tissue microenvironment, while our study assessed circulating monocytes and not synovial macrophages or more in general monocyte-derived cells. Therefore, our finding should not be interpreted as evidence of a specific M1/M2 macrophage polarization state; rather it might indicate an altered monocyte availability that could contribute to the subsequent composition and functional state of tissue macrophages (51). In the T cell subset, a profile predisposed to tissue restoration rather than terminal destruction was apparent: CM cells retain high proliferative capacity and plasticity, which could allow migration to the joint promoting localized tissue repair when stimulated by the growth factors in PRP. Lower baseline Tregs in responders might indicate a different baseline inflammatory endotype, as severe OA often drives compensatory systemic increase in Tregs (52). Finally, lower peripheral abundance of NK cells suggests a less hostile systemic environment, which allows the anabolic and anti-inflammatory properties of PRP to work unhindered.

Additionally, CRUSTY profiling of immunological populations confirmed differential cellular clustering between responders and non-responders. Interestingly, despite a lower frequency of conventionally defined intermediate monocytes in responders (**Figure 2B**), unsupervised analysis identified an enrichment of CD14^+^CD16^+^CD11c^high^HLA-DR^high^ phenotypic clusters (**Figure 3**). This apparent discrepancy may reflect the heterogeneity of the intermediate-monocyte compartment and the limitations of defining monocyte states solely according to CD14 and CD16 expression, as observed in other pathological contexts and by means of a different technology (i.e. CyTOF mass cytometry) (53, 54). In knee OA, responders might exhibit both quantitative differences in monocyte subsets and qualitative changes in their phenotypic composition. As the clustering analysis was performed in the broader cellular compartment rather than specifically within monocytes, this interpretation remains exploratory and warrants confirmation in dedicated monocyte-focused studies. Nonetheless, this evidence supports the hypothesis that the composition of PBMCs at baseline could be used to predict patient responsiveness to PRP therapy for knee OA.

Finally, we conducted a univariate and multivariate analysis of serological parameters and used those that reached statistical significance in the multivariate analysis to define a model able to identify baseline immune characteristics associated with response, for future validation and exploitation as predictors of response; the best ROC curve included IL6 and GROα and had an AUC of 0.845. In the event of future clinical application of the model, quantifying these two specific parameters in a patient being considered for PRP injection might be sufficient to determine the likelihood of response and consequently the appropriateness of the treatment.

This study has some limitations: the overall size of the patient cohort was small (exact number of individuals for each subset of patients and measured parameter are indicated in **Supplementary Table 2**); this did not allow us to perform subanalysis for a stronger statistical power. Also, the large number of comparisons could have led to a type I error in the interpretation of the results. The characterization of the patients was targeted (albeit broad); this may have led to overlooking other pathways and molecules contributing to the response to PRP injection. The kit adopted for PRP preparation was consistent for all patients enrolled in this study, but, since treatment response may depend on the PRP kit used, as already mentioned, we cannot exclude that if using another kit, the composition of the responder and non-responder groups might have been different. The adopted definition of treatment response was mainly based on patient-reported outcomes related to pain and physical function. Finally, despite this study extended the follow-up duration to 12 months, outcomes such as radiographic progression or the need for prosthetic surgery were not evaluated as they would require longer follow-up periods.

Despite these limitations, this work presents an in-depth analysis on a large cohort of knee OA patients before treatment with autologous PRP injection. The multimodal analysis showed significant differences in the expression of inflammatory molecules, markers of oxidative stress, lymphocytic subpopulations between responders and non-responders, for a total follow-up period of 12 months after PRP injection for knee OA.

The next step of this work is the validation of our findings in a larger independent cohort. Furthermore, we foresee to verify whether the panel of markers identified here remain valid irrespective of the kit for PRP preparation. If the results shown here are confirmed, our research will define baseline predictors of response to treatment and contribute to establish precision medicine approaches in knee OA patients that require conservative management.

## Data Availability

The raw data supporting the conclusions of this article will be made available by the authors upon reasonable request.

## Supplementary material

Supplementary Figure 1 Gating strategy applied in the multiparametric FACS analysis. Red arross point to cell subsets that were more abundant in responders, while blue arrows point to subsets that were less abundant in responders compared to non-responders.
