# Supplementary material for: Immuno-based profiling of knee osteoarthritis patients identifies baseline characteristics associated with positive response to autologous platelet-rich plasma injection at one-year follow-up
Source: Front Immunol. 2026 Sep 4;17:1951258. doi: 10.3389/fimmu.2026.1951258 (PMC13585714; doi:10.3389/fimmu.2026.1951258)
Supplement: Supplementary file 1 [file Table1.docx]

**Immuno-based profiling of knee osteoarthritis patients predicts the response to autologous platelet-rich plasma injection at one-year follow-up**

Valentina Granata^1,*^, Antonio Tonutti^2,3,*^, Veronica Marrella^4,5^, Chiara Camisaschi^6^, Simone Puccio^4,7^, Cristiano Sconza^8^, Marzia Monferini^3^, Nicola Rani^9^, Giuseppe Filardo^10^, Berardo Di Matteo^2,11^, Carlo Selmi^2,3^, Emanuela Morenghi^2,12^, Cristina Sobacchi^4,13,§^, Angela Ceribelli^2,14^

**Supplementary Material**

**Supplementary Table 1.** Demographic and clinical characteristics of the enrolled patients, divided into based on the response one year after intra-articular PRP injection. ^a^Mann-Whitney test; ^b^Chi-square

|  | **Total patients**  (n = 109) | **Responders**  (n = 60) | **Non-Responders**  (n = 49) | **p value** |
| --- | --- | --- | --- | --- |
| **Age**  *years* (range) | 58.6  (40-74) | 56.5  (40-74) | 59  (42-74) | 0.32^a^ |
| **Sex** (M:F) | 46:63 | 28:32 | 18:31 | 0.29^b^ |
| **Symptoms durations**  *months* (range) | 12  (1-420) | 12  (1-420) | 24  (6-168) | 0.031^a^ |
| **Previous surgery** (no:yes) | 88:21 | 51:9 | 37:12 | 0.21^b^ |

**Supplementary Table 2.** Median, ranges and statistics of the parameters measured in our cohort; the exact number of subjects is indicated, too, for each readout.

|  | **n** | **Total patients**  **(n=109)** | **n** | **Responders**  **(n = 60)** | **n** | **Non-Responders**  **(n = 49)** | **p value** |
| --- | --- | --- | --- | --- | --- | --- | --- |
| **Antioxidant capacity** (μM) | 109 | 405 (214 – 952) | 60 | 407 (265 – 840) | 49 | 390 (214 – 952) | 0.013 |
| **Vitamin E** (μg/ml) | 107 | 14.1 (0.1 – 76.3) | 59 | 17.4 (0.7 – 67.1) | 48 | 8.5 (0.1 – 76.3) | 0.093 |
| **4-HNE** (ng/ml) | 106 | 59.6 (0.4 – 675.1) | 59 | 45.1 (0.8 – 212.8) | 47 | 77.2 (0.4 – 675.1) | 0.022 |
| **TNFα** (pg/ml) | 75 | 12.2 (0.2 – 164.0) | 52 | 11.7 (0.2 – 104.0) | 23 | 15.8 (0.4 – 164.0) | 0.002 |
| **IL-10** (pg/ml) | 75 | 2.2 (0.1 – 798.0) | 52 | 2.1 (0.1 – 25.1) | 23 | 2.7 (0.3 – 798.0) | 0.083 |
| **IL-6** (pg/ml) | 75 | 2.9 (0.1 – 122.0) | 52 | 2.8 (0.4 – 11.8) | 23 | 3.2 (0.1 – 122.0) | 0.220 |
| **PTX3** (ng/ml) | 105 | 170 (0 – 1885410) | 58 | 117 (0 – 15520) | 47 | 910 (24 – 1885410) | <0.001 |
| **CD40L** (pg/ml) | 108 | 3676 (948 – 10470) | 60 | 3781 (948 – 6634) | 48 | 3225 (984 – 10470) | 0.030 |
| **Eotaxin** (pg/ml) | 109 | 160 (10 – 525) | 60 | 170 (10 – 465) | 49 | 156 (10 – 525) | 0.058 |
| **G-CSF** (pg/ml) | 109 | 27.0 (3.2 – 100.8) | 60 | 30.4 (3.2 – 46.4) | 49 | 21.8 (7.3 – 100.8) | 0.035 |
| **GROα** (pg/ml) | 99 | 120 (13.6 – 360) | 57 | 130 (50 – 298) | 42 | 105 (13.6 – 360) | 0.024 |
| **MIP3b** (pg/ml) | 109 | 51.7 (15.8 – 967.9) | 60 | 59.5 (20.4 – 402.4) | 49 | 48.3 (15.8 – 967.9) | 0.026 |
| **PDGF-AA** (pg/ml) | 89 | 3648 (285 – 10832) | 52 | 2898 (285 – 10463) | 37 | 5894 (532 – 10832) | 0.004 |
| **PD-L1** (pg/ml) | 109 | 77.9 (14.5 – 218.3) | 60 | 83.2 (19.9 – 193.3) | 49 | 66.6 (14.5 – 218.3) | 0.054 |
| ***IL1α*** (AU) | 78 | 9.4 (0.5 – 453.9) | 41 | 7.7 (0.5 – 453.9) | 37 | 11.1 (0.5 – 384.0) | 0.020 |
| ***IL4*** (AU) | 105 | 1.1 (0.04 – 43.9) | 57 | 0.9 (0.04 – 14.9) | 48 | 1.4 (0.1 – 43.9) | 0.013 |
| ***IL17A*** (AU) | 103 | 5.99 (0.1 – 492.1) | 56 | 4.75 (0.50 – 492.1) | 47 | 9.35 (0.10 – 399.2) | 0.021 |
| ***MYD88*** (AU) | 107 | 3.0 (0.0 – 37.0) | 58 | 4.5 (0.04 – 37.0) | 49 | 0.7 (0.0 – 20.8) | <0.001 |
| ***PTGS2*** (AU) | 104 | 10.1 (0.2 – 893.8) | 56 | 8.4 (0.2 – 76.7) | 48 | 11.2 (1.4 – 893.8) | 0.013 |
| ***IL36RA*** (AU) | 105 | 1.9 (0.03 – 765.7) | 56 | 1.5 (0.1 – 50.2) | 49 | 3.4 (0.03 – 765.7) | 0.011 |
| ***IL36α*** (AU) | 105 | 0.55 (0.001-7.05) | 57 | 0.58 (0.001-5.38) | 48 | 0.66 (0.08-7.05) | 0.043 |
| ***IL36γ*** (AU) | 104 | 0.38 (0.003-228.5) | 56 | 0.29 (0.003-228.5) | 48 | 0.58 (0.007-103.2) | 0.03 |
| ***CCL3*** (AU) | 107 | 148.8 (7.85-26508) | 58 | 105.6 (7.85-3222) | 49 | 244.4 (15,73-26508) | 0.012 |

**Supplementary Table 3.** Univariate and multivariate analysis.

|  | **Univariable** |  | **Multivariable** |  |
| --- | --- | --- | --- | --- |
|  | **OR (95% CI)** | **p value** | **OR (95% CI)** | **p value** |
| **Sex (M)** | 1.51 (0.70 – 3.26) | 0.297 |  |  |
| **Age** | 0.98 (0.93 – 1.03) | 0.368 |  |  |
| **Symptoms duration** | 1.00 (0.99 – 1.01) | 0.600 |  |  |
| **Previous surgery** | 0.54 (0.21 – 1.42) | 0.215 |  |  |
| **Antioxidant capacity** | 1.004 (1.000 – 1.008) | 0.029 |  |  |
| **Vitamin E** | 1.01 (0.99 – 1.04) | 0.288 |  |  |
| **4-HNE** | 0.992 (0.987 – 0.998) | 0.008 |  |  |
| **TNFα** | 0.97 (0.95 – 0.99) | 0.013 |  |  |
| **IL-10** | 0.99 (0.86 – 1.13) | 0.857 |  |  |
| **IL-6** | 0.88 (0.78 – 0.99) | 0.040 | 0.82 (0.69 – 0.97) | 0.024 |
| **PTX3** | 1.000 (1.000 – 1.000) | 0.066 |  |  |
| **CD40L** | 1.000 (1.000 – 1.000) | 0.113 |  |  |
| **Eotaxin** | 1.003 (0.999 – 1.007) | 0.089 |  |  |
| **G-CSF** | 1.02 (0.99 – 1.05) | 0.222 |  |  |
| **GROα** | 1.01 (1.00 – 1.01) | 0.055 | 1.02 (1.01 – 1.05) | 0.004 |
| **MIP3b** | 0.999 (0.996 – 1.003) | 0.794 |  |  |
| **PDGF-AA** | 1.000 (1.000 – 1.000) | 0.008 |  |  |
| **PD-L1** | 1.01 (1.00 – 1.02) | 0.139 |  |  |
